# NO-Dependent Mechanisms of p53 Expression and Cell Death in Rat’s Dorsal Root Ganglia after Sciatic-Nerve Transection

**DOI:** 10.3390/biomedicines10071664

**Published:** 2022-07-11

**Authors:** Stanislav Rodkin, Valentina Dzreyan, Mikhail Bibov, Alexey Ermakov, Tatyana Derezina, Evgeniya Kirichenko

**Affiliations:** 1 Faculty of Bioengineering and Veterinary Medicine, Don State Technical University, Gagarin Square 1, 344000 Rostov-on-Don, Russia; amermakov@ya.ru (A.E.); derezinasovet@mail.ru (T.D.); kiriche.evgeniya@yandex.ru (E.K.); 2 Laboratory of Molecular Neurobiology, Academy of Biology and Biotechnology, Southern Federal University, Stachki Ave., 194/1, 344090 Rostov-on-Don, Russia; dzreyan2016@mail.ru; 3 Department of General and Clinical Biochemistry No. 2, Rostov State Medical University, Nakhichevansky, 29, 344022 Rostov-on-Don, Russia; mbibov@gmail.com

**Keywords:** axotomy, dorsal root ganglion, nitric oxide, inducible NO-synthase, apoptosis, p53

## Abstract

Peripheral-nerve injury is a frequent cause of disability. Presently, no clinically effective neuroprotectors have been found. We have studied the NO-dependent expression of p53 in the neurons and glial cells of the dorsal root ganglia (DRG) of a rat’s spinal cord, as well as the role of NO in the death of these cells under the conditions of axonal stress, using sciatic-nerve axotomy as a model. It was found out that axotomy led to the nuclear–cytoplasmic redistribution of p53 in neurons, 24 h after trauma. The NO donor led to a considerable increase in the level of p53 in nuclei and, to a smaller degree, in the cytoplasm of neurons and karyoplasm of glial cells 4 and 24 h after axotomy. Application of a selective inhibitor of inducible NO-synthase (iNOS) provided the opposite effect. Introduction of the NO donor resulted in a significant increase in cell death in the injured ipsilateral DRG, 24 h and 7 days after trauma. The selective inhibitor of iNOS demonstrated a neuroprotective effect. Axotomy was shown to upregulate the iNOS in nuclei and cytoplasm of DRG cells. The NO-dependent expression of p53, which is particularly achieved through iNOS activation, is believed to be a putative signaling mechanism of neural and glial-cell death after axotomy.

## 1. Introduction

Neurotrauma causes millions of deaths annually throughout the world [1]. It is considered to be a challenging medical problem because of the absence of clinically effective neuroprotective medications. About 10% of neurotrauma is connected with peripheral-nerve injury, which can be up to total disruption that often leads to disability [2,3]. One of the types of mechanical injury to nervous tissue is axotomy, which is characterized by axon transection, resulting in either neuron death or sensory and motor recovery. An important condition of neuron survival in this pathological process is intercellular interaction with glial cells [4].

Signaling processes involved in the survival or death of neurons and glial cells under axotomy conditions are poorly studied, so their exploration is believed to be fundamentally and practically important for further investigations. Thereby, study of the role of nitrogen oxide (NO) is considered to be of special interest, since it serves as a universal messenger, which fulfills a number of different functions in the organism, including modulation of a variety of processes in the nervous tissue under both normal and pathological conditions. It is known that NO may be involved in neuron regeneration after an axon injury [5]. However, its role in recovery after axotomy is contradictory, and NO-dependent signaling pathways aimed at the regulating of survival and death of neurons and glial cells remain unclear. It was shown that NO works as a negative regulator under the conditions of axotomy, which leads to the degradation and death of injured neurons [6,7,8]. Other studies, however, showed a neuroprotective effect of NO under the axonal-stress conditions [5,9,10,11]. A question about the roles of different NO-synthase (NOS) isoforms, the enzymes, which are responsible for NO biosynthesis, in neurons after injury of the nerve, also remains a matter for discussion.

Presently, three isoforms of NOS are known. These are endothelial and neuronal NOS (eNOS/NOS3, nNOS/NOS1), which belong to the constitutive Ca^2+^-dependent isoforms of NOS, and the inducible Ca^2+^-independent NOS (iNOS/NOS2) [12]. The latter is capable of generating more than 10 times higher levels of NO as compared with eNOS and nNOS and, therefore, may provide a stronger effect on cells and is considered to be of special interest [13]. It was shown that sciatic-nerve transection induces upregulation of all NOS isoforms, including the iNOS [14].

Apart from the classical NO-activated signaling pathway sGC/cGMP/PKG, NO is involved into other molecular cellular signaling cascades [15]. It was found out that NO may induce accumulation of p53 protein, a well-known tumor suppressor and “genome guard” [16,17,18]. The p53 protein regulates a number of important cellular functions, including DNA repair, the cell cycle, metabolism, and apoptosis [19,20,21]. It also plays an important role in neuropathological processes induced by ischemia, oxidative stress, cancer, and neurodegenerative diseases [22,23]. Several studies point to the connection between p53 upregulation and neuron death from an injury to the nerves [24,25,26]. Some other authors reported about the neuroprotector effect of p53, which manifested as the regeneration of damaged axons [27]. Our previous studies revealed the increase in the level of p53 in mechanoreceptor neurons and glial cells of crayfish after axotomy [28], as well as in the dorsal root ganglia (DRG) of a rat’s spinal cord after sciatic-nerve transection [29]. However, signaling mechanisms, which regulate the level of p53 after axotomy, remain unclear and require further investigations.

The goal of this work was to study the NO-dependent signaling pathways, which regulate the expression of p53 in neurons and glial cells of DRG, as well as to assess the role of NO in death of these cells under the conditions of axonal stress induced by sciatic-nerve transection.

## 2. Materials and Methods

### 2.1. Animals and Ethical Approval

The experiments with sciatic-nerve transection were carried out with adult male rats, Wistar line, weight 200–250 g, which were kept in standard cages in groups of 4–5 animals with free access to food and water. All animals were kept under similar standard conditions: 12 h light/12 h dark, room temperature of 22–25 °C, and the ventilation rate of 18 air changes per hour. Animals were kept and treated in accordance with all international, national, and/or institutional regulations. All experimental procedures with animals were carried out in accordance with the EU regulations 86/609/EEC and local law for experimental ethics. The experimental protocols were assessed and approved by the Committee for Animal Treatment of Southern Federal University (Approval No. 08/2016).

### 2.2. Objects and Procedure of Axotomy

DRGs contain sensor-neuron bodies. Axons of the DRG neurons are included in the sciatic nerve, which is responsible for innervation of hind limbs. Rats were anesthetized intramuscularly by injection of 0.75 mL of 2:1 mixture of Xyla (25 xylazine hydrochloride, Interchemie Werken “de Adelaar” BV, St. Waalre, The Netherlands) and telazol (mixture of Tiletamine hydrochloride and zolazepam hydrochloride, Zoetis, St. Kalamazoo, MI, USA). The operation of sciatic-nerve transection and DRG isolation was performed as described in Savastano et al. [30]. Undamaged contralateral DRGs were used as a control. Rats were anesthetized and decapitated with guillotine 4 h, 24 h, and 7 days after unilateral transection of the sciatic nerve.

The study of NO-dependent mechanisms of regulation of p53 expression, as well as the study of the effects of NO on neural and glial-cell survival and death in DRG after sciatic-nerve transection, were performed using the following NO-modulators: the iNOS selective inhibitor S-methylisothiourea hemisulfate (SMT) and the NO donor Sodium nitroprusside, which were obtained from Sigma-Aldrich (St. Louis, MO, USA). Both SMT (10 mg/kg) [31] and SNP (3 mg/kg) [32] were administered immediately after sciatic-nerve transection and further administered daily until decapitation. The control group of animals was treated with a physiological solution instead of modulators. The study also included intact animals, which did not undergo sciatic-nerve transection and were treated with physiological solution until decapitation.

Groups of rats, which consisted of 6 and 18 animals, were used for fluorescence microscopy and Western blot, respectively.

All animals were selected blindly without any assessment of appearance and behavior and divided randomly into groups.

### 2.3. Immunofluorescent Microscopy

To reveal the expression of p53 in DRG 24 h and 7 days after sciatic-nerve transection, the 4th ganglion was isolated fast and fixed in 4% paraformaldehyde for 6 h and incubated in 20% sucrose for 48 h at 4 °C. Then, the ganglion was embedded into the 4% agarose gel (low-melt agarose, Sigma-Aldrich, St. Louis, MO, USA). Slices of agarose blocks of about 20-μm thickness were obtained with a Leica VT 1000 S vibratome (Leica, St. Heidelberger, Germany), washed, in PBS and incubated in 5% BSA supplemented with 0.3% Triton X-100 for 1 h at room temperature, in order to block the sites of non-specific binding. Next, the slides were incubated with the primary antibody against p53 (P5813, Sigma-Aldrich, St. Louis, MO, USA; 1:100) and the rat antibody against the neuronal specific nuclear protein NeuN (ABN78, Sigma-Aldrich, St. Louis, MO, USA; 1:1000) overnight at 4 °C. Slides were washed thrice with PBS and incubated with secondary antibodies anti-rabbit IgG (H+L), CF™ 488A (SAB4600045, Sigma-Aldrich, St. Louis, MO, USA, 1:500) and the anti-mouse IgG1 (γ1), CF™555 (SAB4600302, Sigma-Aldrich, St. Louis, MO, USA; 1:500). Incubation without primary antibodies was used as the negative control. Nuclei of neurons and glial cells were visualized with Hoechst 33342 (40 μM; 10 min) [33]. Slides were imbedded into 60% glycerol and examined with Olympus BX-51 microscope, equipped with the OrcaFlash 4.0 V3 digital camera (Hamamatsu, St. Iwata, Japan). Colocalization of p53 with the NeuN neuronal marker was assessed with the ImageJ (Rasband, W.S., ImageJ, U.S. National Institutes of Health, Bethesda, MD, USA, https://imagej.nih.gov/ij/ (accessed on 4 October 2021)) software using the JACoP plug-in [34]. The colocalization coefficient M1 mirrors the portion of pixels, which contain both red (p53) and green (NeuN) signals, with respect to the total signal registered in the green channel [35]. All calculations were carried out for at least 100 cells. To perform the quantitative assessment of the mean level of p53-related fluorescence in the experimental and control preparations of DRG, 10 control and 10 experimental images were analyzed for 6 rats. Mean fluorescent areas in cytoplasm and nucleus were obtained for each cell and mean values of the data obtained were calculated.

### 2.4. Western Blot

The expression of p53 and iNOS in the nuclear and cytoplasmic fractions of DRGs of rats, which underwent axotomy, has been studied by Western blot. The 4th and 5th DRGs obtained from tree rats were combined for each time interval after axotomy, in order to obtain sufficient amount of biological material. Hence, each experimental group consisted of samples obtained from at least 18 animals (3 animals per experiment; 6 experiments in total).

Both cytoplasmic and nuclear fractions were obtained using the CelLytic™ NuCLEAR™ Extraction Kit (NXTRACT, Sigma-Aldrich, St. Louis, MO, USA). Samples were homogenized in the Lysis Buffer on ice for 3 min with the Vibra-Cell VCX 130 (Sonics, St. Newtown, CT, USA). The Lysis Buffer was obtained from the CelLytic™ NuCLEAR™ Extraction Kit (NXTRACT, Sigma-Aldrich, St. Louis, MO, USA) and supplemented with protease and phosphatase inhibitors (PPC1010, Sigma-Aldrich, St. Louis, MO, USA), in order to preserve proteins and their phosphorylated forms, as well as by nuclease benzonase (E1014, Sigma-Aldrich, St. Louis, MO, USA), which digests nucleic acids. The homogenized samples were centrifuged at 10,000–11,000× *g* at 4 °C for 20 min using the Mikro 220R centrifuge (Hettich, St. Tuttlingen, Germany). The supernatant obtained, which contained cytoplasmic proteins, was collected, and the pellet, which contained nuclei and cellular debris, was used to extract nuclear proteins. The extractions of nuclear proteins were carried out with the Nuclear Extraction Buffer, a part of the NXTRACT kit. Briefly, the pellet was resuspended and incubated in the Nuclear Extraction Buffer for 40 min. The lysate obtained was centrifuged at 20,000–21,000× *g* for 5 min at 40 °C. The cytoplasmic fraction was confirmed by the absence of the H4 histon. The protein level in the extracts obtained was measured by the Bradford method (B6916, Sigma Aldrich, St. Louis, MO, USA).

Samples, which contained 10–20 μg of protein in 15 μL, were analyzed by electrophoresis in 7–10% SDS-polyacrylamide gel in the mini-PROTEAN Tetra electrophoretic cell (Bio-Rad, St. Hercules, CA, USA). The ColorBurst Electrophoresis Marker (C1992, Sigma-Aldrich, St. Louis, MO, USA) was used as a molecular-weight standard. After electrophoresis, proteins were transferred onto the PVDF-membrane (polyvinyl difluoride membrane 162-0177, Bio-Rad, St. Hercules, CA, USA) using the Trans-Blot^®^ Turbo Transfer System (Bio-Rad, St. Hercules, CA, USA). The membrane was washed with PBS and blocked in TBS 1% Casein Blocker (Bio-Rad, St. Hercules, CA, USA) for 1 h. Then, the membrane was incubated with mice primary antibodies against either p53 (1:500) (P5813, Sigma-Aldrich, St. Louis, MO, USA) or iNOS (1:500) (N7782, Sigma-Aldrich, St. Louis, MO, USA) or Bax (SAB5700071, Sigma-Aldrich, St. Louis, MO, USA), plus Bcl-2 (SAB5700676, Sigma-Aldrich, St. Louis, MO, USA), as well as with antibodies against β-actin (A5441, 1:5000, Sigma-Aldrich, St. Louis, MO, USA) in both cases, overnight at 4 °C. Next, the membranes were washed with 10 mM Tris-buffer and pH 8.0 supplemented with 0.1% Tween-20 (TTVS) and incubated with rabbit secondary IgG-peroxidase antibodies (1:1000) (A6154, Sigma-Aldrich, St. Louis, MO, USA) and mice IgG-peroxidase antibodies (1:1000) (A4416-1ML, Sigma-Aldrich, St. Louis, MO, USA) for 1 h at room temperature. The Western blots obtained were visualized with the Clarity Western ECL Substrate (Bio-Rad, St. Hercules, CA, USA), using the Fusion SL gel documentation system (Vilber Lourmat, Collegien, France). The images obtained were processed with the Vision Capt software (Vilber Lourmat, France, https://visioncapt.software.informer.com/ (accessed on 4 October 2021)).

### 2.5. The Analysis of Cell Death

Cell death was determined using the “In Situ Cell Death Detection Kit, TMR red” (Sigma-Aldrich), in which single-strand DNA breaks are marked by the TUNEL method (Transferase-mediated dUTP Nick-End Labeling). 

The reaction mixture was prepared ex tempore before use and kept on ice throughout the experiment. Slices of DRGs were fixed with 4% paraformaldehyde, transferred on microscope slides, washed with PBS thrice, and permeabilized by incubation in 0.1% sodium citrate supplemented with 0.1% Triton X-100 (Sigma-Aldrich, St. Louis, MO, USA) for 2 min at 4 °C. After permeabilization, slides were washed with PBS twice and dried. Each slide was treated with 50 μL of TUNEL reagent (Sigma-Aldrich, St. Louis, MO, USA) and incubated in a humid chamber in the dark for 60 min at 37 °C.

After incubation, slides were washed with PBS three times, dried, and embedded into glycerol. Positive control was performed by 10 min incubation of permeabilized slides with nuclease benzonase (1500 U/mL in 50 mM Tris-HCl buffer, pH 7.5, which contained 1 mg/mL BSA) at 25 °C, in order to assess the efficacy of neuron and glial cell apoptosis analysis in DRG sections by TUNEL. Slides were contrastingly stained with Hoechst 33,342 (40 μM, 10 min) that allowed visualization of neural and glial cell nuclei [33]. The preparations obtained were analyzed with an Olympus BX51WI microscope (Olympus, Tokyo, Japan) equipped with the ORCA-Flash4.0 V3 digital camera at a 570–620-nm excitation wavelength. The TUNEL-positive cells were counted in 3 sections of the 4th DRGs, both intact and the one that underwent axotomy. The DRGs were obtained from 6 animals of each experimental group, which were treated with either physiological solution, SMT, or SNP, 24 h and 7 days after sciatic-nerve transection.

### 2.6. The Statistical Analysis

The statistical analysis was performed by the one-way analysis of variance (ANOVA) with a Dunnett’s post hoc test. The normality and homogeneity of dispersion was assessed by the Shapiro–Wilk and Brown–Forsythe tests, respectively. If the normality or homogeneity of dispersion were not confirmed, then it was assessed by non-parametric Kruskal–Wallis H-test. All study results were analyzed on a blind basis. Differences were considered confident at *p* < 0.05 and *n* = 6. The data obtained are expressed as mean ± SEM.

## 3. Results

### 3.1. Nuclear-Cytoplasmic Redistribution of p53 and NO-Dependent Deposition of p53 in Neurons and Glial Cells after Axotomy

It is known that NO affects the expression and localization of p53 in different cells after stress [17,18,36,37,38,39]. However, mechanisms of NO-dependent regulations of the level of p53 in neurons and glial cells after axotomy are scarcely studied. To assess the role of NO in expression and localization of p53 after axotomy, we performed the immunofluorescent and Western blot analyses of DRG cells, which were obtained from rats treated with physiological solution, NO donor, or the selective inhibitor of iNOS after sciatic-nerve transection.

**Figure 1:**
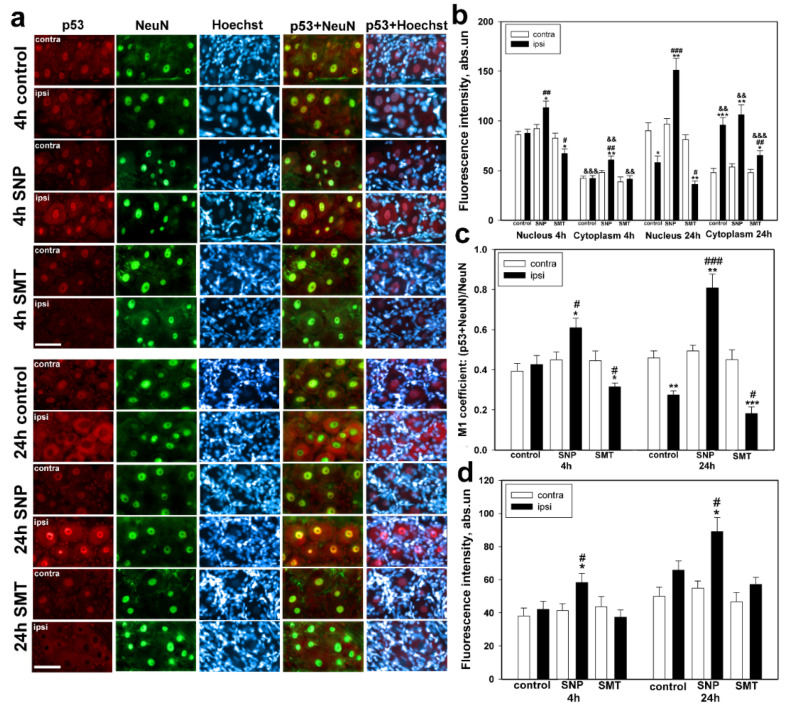
Immune-fluorescent microscopy. (**a**) Expression of p53 (red fluorescence) in DRG neurons of rats of the control group, which were treated with physiological solution, and the experimental group, which were treated with either the NO donor (SNP) or the iNOS selective inhibitor (SMT), 4 h and 24 h after sciatic-nerve transection. The scale section is 50 μm. (**b**) The dependence of mean p53 fluorescence intensity in the nuclei and cytoplasm of neurons of the contralateral and ipsilateral DRG of the control and experimental groups of animals 4 h and 24 h after sciatic-nerve transection. (**c**) The M1 coefficient of colocalization of p53 and the NeuN neuronal nuclei marker in the contralateral and ipsilateral DRGs of the control and experimental groups of animals 4 h and 24 h after sciatic-nerve transection. (**d**) The dependence of mean p53 fluorescence intensity in glial cell nuclei of DRGs 4 h and 24 h after sciatic-nerve transection. Ipsi—the axotomized ipsilateral ganglion; contra—the contralateral ganglion; NeuN—neuronal nuclei marker (green fluorescence); NeuN+p53 and Hoechst+p53—overlap; Hoechst—the fluorescence of Hoechst 33,342, which allows visualization of nuclei of both neurons and glial cells. One-way ANOVA. Mean ± SEM. *n* = 6. * *p* < 0.05, ** *p* < 0.01, *** *p* < 0.001—ipsilateral DRG with respect to the contralateral DRG of the same animal; # *p* < 0.05, ## *p* < 0.01, ### *p* < 0.001—ipsilateral DRG with respect to the ipsilateral DRG of the control group animal within the framework of the same time interval after axotomy; && *p* < 0.01, &&& *p* < 0.001—ipsilateral DRG with respect to the ipsilateral DRG of the same animal.

The immune-fluorescent microscopy showed that p53 is located in the DGR neurons and glial cells, the nuclei of which were visualized with Hoechst 33342 [33] (Figure 1a). It was shown that the level of pro-apototic p53 protein changed differently in neurons of injured ipsilateral DRGs in comparison with contralateral DRGs, which did not undergo sciatic-nerve transection. For example, the redistribution of p53 between the nucleus and cytoplasm was observed in neurons of the control group of animals, which underwent axotomy and received physiological solution 24 h after trauma (Figure 1a,b).

However, 4 h after trauma, the level of p53 in both nuclei and cytoplasm of neurons of damaged DRGs was not different from that in the control samples. The p53-related fluorescence was revealed mainly in the neuron nucleoplasm, and it was twice as high as that in the cytoplasm (one-way ANOVA with a Dunnett’s post hoc test, *p* < 0.001, F_1,10_ = 75.156, Shapiro–Wilk test *p* = 0.316, Brown–Forsythe test *p* = 0.355). Similar ratios of fluorescence were observed in the contralateral DRGs as well (one-way ANOVA with a Dunnett’s post hoc test, *p* < 0.001, F_1,10_ = 114.784, Shapiro–Wilk test *p* = 0.887, Brown–Forsythe test *p* = 0.118) (Figure 1b). In 24 h after axotomy the immune fluorescence of p53 in the nuclei of neurons, which underwent axotomy decreased by 1.6 times (one-way ANOVA with a Dunnett’s post hoc test, *p* < 0.05, F_1,10_ = 9.450, Shapiro–Wilk test *p* = 0.961, Brown–Forsythe test *p* = 0.818), whereas in the cytoplasm it increased almost twice (one-way ANOVA with a Dunnett’s post hoc test, *p* < 0.001, F_1,10_ = 30.442, Shapiro–Wilk test *p* = 0.859, Brown–Forsythe test *p* = 0.360) in comparison with the contralateral DRG obtained from the same animal (Figure 1b). Translocation of p53 from the nucleus into the cytoplasm of ipsilateral DRGs 24 h after axotomy was confidently confirmed by the decrease in the M1 coefficient, which characterizes colocalization of p53 with the NeuN neuronal nuclei marker, by 40%, with respect to the contralateral ganglia (one-way ANOVA with a Dunnett’s post hoc test, *p* < 0.01, F_1,10_ = 20.441, Shapiro–Wilk test = 0.285, Brown–Forsythe test *p* = 0.180) (Figure 1c).

The administration of the NO donor SNP led to the increase in the p53 level in both neuronal nuclei by 23% (one-way ANOVA with a Dunnett’s post hoc test, *p* < 0.05, F_1,10_ = 7.103, Shapiro–Wilk test *p* = 0.391, Brown–Forsythe test *p* = 0.057) and 56% (one-way ANOVA with a Dunnett’s post hoc test, *p* < 0.01, F_1,10_ = 16.800, Shapiro–Wilk test *p* = 0.122, Brown–Forsythe test *p* = 0.284) and in the cytoplasm by 27% (one-way ANOVA with a Dunnett’s post hoc test, *p* < 0.05, F_1,10_ = 7.510, Shapiro–Wilk test *p* = 0.8, Brown–Forsythe test *p* = 0.053) and 98% (Kruskal–Wallis H-test, *p* < 0.01, Shapiro–Wilk test *p* < 0.05), respectively, 4 h and 24 h after axotomy, with respect to the values obtained for the contralateral ganglion of the same animal (Figure 1a,b).

It was shown that 4 h and 24 h after axotomy, the absolute fluorescence of p53 was higher in the nuclear area than in the cytoplasm of the ipsilateral DRG neurons. The comparative analysis of p53-related fluorescence in the nuclei and cytoplasm of neurons of animals, which underwent axotomy and received SNP, 4 h and 24 h after, with respect to the ipsilateral ganglia of the control group, revealed an increase in the fluorescence by 29% (one-way ANOVA with a Dunnett’s post hoc test, *p* < 0.01, F_1,10_ = 10.702, Shapiro–Wilk test *p* = 0.326, Brown–Forsythe test *p* = 0.08) and an almost three-time increase (one-way ANOVA with a Dunnett’s post hoc test, *p* < 0.001, F_1,10_ = 45.694, Shapiro–Wilk test *p* = 0.264, Brown–Forsythe test *p* = 0.375) in the nuclei, respectively, whereas in the cytoplasm a 40% increase was observed 4 h after trauma (one-way ANOVA with a Dunnett’s post hoc test, *p* < 0.01, F_1,10_ = 13.094, Shapiro–Wilk test *p* = 0.457, Brown–Forsythe test *p* = 0.260) (Figure 1b).

The NO-dependent accumulation of p53 observed in the nuclei of neurons, which underwent axotomy, was confirmed by the M1 coefficient. Values of p53 and NeuN in the DRG of rats, which underwent axotomy, and in the control DRG, to which SNP was administered, increased by 33% (one-way ANOVA with a Dunnett’s post hoc test, *p* < 0.05, F_1,10_ = 6.726, Shapiro–Wilk test *p* = 0.571, Brown–Forsythe test *p* = 0.817) and 63% (one-way ANOVA with a Dunnett’s post hoc test, *p* < 0.01, F_1,10_ = 17.667, Shapiro–Wilk test *p* = 0.681, Brown–Forsythe test *p* = 0.191), respectively, 4 h and 24 h after trauma. The comparative analysis of the M1 coefficient obtained for the ipsilateral DRGs of animals, treated with SNP, was increased by 36% (one-way ANOVA with a Dunnett’s post hoc test, *p* < 0.05, F_1,10_ = 7.964, Shapiro–Wilk test *p* = 0.1, Brown–Forsythe test *p* = 0.980) and by almost three times (one-way ANOVA with a Dunnett’s post hoc test, *p* < 0.001, F_1,10_ = 54.752, Shapiro–Wilk test *p* = 0.478, Brown–Forsythe test *p* = 0.120) in comparison with the control ipsilateral DRG, 4 h and 24 h after axotomy, respectively (Figure 1c).

The opposite dynamics was observed in the ipsilateral DRGs of the experimental group animals, which were treated with SMT. It was shown to be similar to the dynamics of the control group, in which redistribution of p53 between nucleus and cytoplasm was observed in neurons, which underwent axotomy. For example, the level of p53 in the nucleus of ipsilateral DRG neurons of rats treated with SMT was decreased by 20% (one-way ANOVA with a Dunnett’s post hoc test, *p* < 0.05, F_1,10_ = 5.051, Shapiro–Wilk test *p* = 0.495, Brown–Forsythe test *p* = 0.392) and 55% (one-way ANOVA with a Dunnett’s post hoc test, *p* < 0.01, F_1,10_ = 60.109, Shapiro–Wilk test *p* = 0.773, Brown–Forsythe test *p* = 0.781), 4 h and 24 h after axotomy, as compared with the contralateral DRG of the same animal (Figure 1b). In the cytoplasm of axotomized neurons a significant increase in the level of p53 was observed 24 h after axotomy, as compared with the contralateral ganglion. However, in comparison with the ipsilateral DRG of the control group, this increase was higher by 37% (one-way ANOVA with a Dunnett’s post hoc test, *p* < 0.01, F_1,10_ = 11.875, Shapiro–Wilk test *p* = 0.933, Brown–Forsythe test *p* = 0.448) lower (Figure 1b). The p53-related fluorescence in the karyoplasm of the ipsilateral DRG neurons of rats, which were treated with SMT, was decreased by 23% (one-way ANOVA with a Dunnett’s post hoc test, *p* < 0.05, F_1,10_ = 10.081, Shapiro–Wilk test *p* = 0.372, Brown–Forsythe test *p* = 0.879) and 37% (one-way ANOVA with a Dunnett’s post hoc test, *p* < 0.05, F_1,10_ = 8.330, Shapiro–Wilk test *p* = 0.186, Brown–Forsythe test *p* = 0.443), 4 h and 24 h after axotomy, respectively, in comparison with the ipsilateral DRGs of animals of the control group (Figure 1b). Values of the p53 and NeuN colocalization in the axotomized ganglia were decreased by 29% (one-way ANOVA with a Dunnett’s post hoc test, *p* < 0.05, F_1,10_ = 6.485, Shapiro–Wilk test *p* = 0.253, Brown–Forsythe test *p* = 0.242) and 60% (Kruskal–Wallis H-test, *p* < 0.01, Shapiro–Wilk test *p* < 0.05), 4 h and 24 h after axotomy, respectively, as compared with the undamaged DRGs of the experimental animals, which were treated with SMT. The M1 coefficient of the ipsilateral DRGs of animals treated with SMT was decreased by 26% (one-way ANOVA with a Dunnett’s post hoc test, *p* < 0.05, F_1,10_ = 5.278, Shapiro–Wilk test *p* = 0.745, Brown–Forsythe test *p* = 0.118) and 33% (one-way ANOVA with a post hoc Dunnett’s test, *p* < 0.05, F_1,10_ = 5.503, Shapiro–Wilk test *p* = 0.306, Brown–Forsythe test *p* = 0.509), 4 h and 24 h after axotomy, respectively, in comparison with the ipsilateral DRGs of the control group (Figure 1c).

In glial cells p53 was localized mostly in the nuclei. Introduction of NO induced upregulation of p53 in glial cells of the axotomized ganglia with respect to both contralateral DRG (one-way ANOVA with a Dunnett’s post hoc test, *p* < 0.05, F_1,10_ = 5.952, Shapiro–Wilk test *p* = 0.245, Brown–Forsythe test *p* = 0.717 and Kruskal–Wallis H-test *p* < 0.05, Shapiro–Wilk test *p* < 0.05) of the same animal and the ipsilateral DRGs of the control group (one-way ANOVA with a Dunnett’s post hoc test, *p* < 0.05, F_1,10_ = 5.013, Shapiro–Wilk test *p* = 0.176, Brown–Forsythe test *p* = 0.809 and Kruskal–Wallis H-test, *p* < 0.05, Shapiro–Wilk test *p* < 0.05), 4 and 24 h after axotomy (Figure 1d).

**Figure 2:**
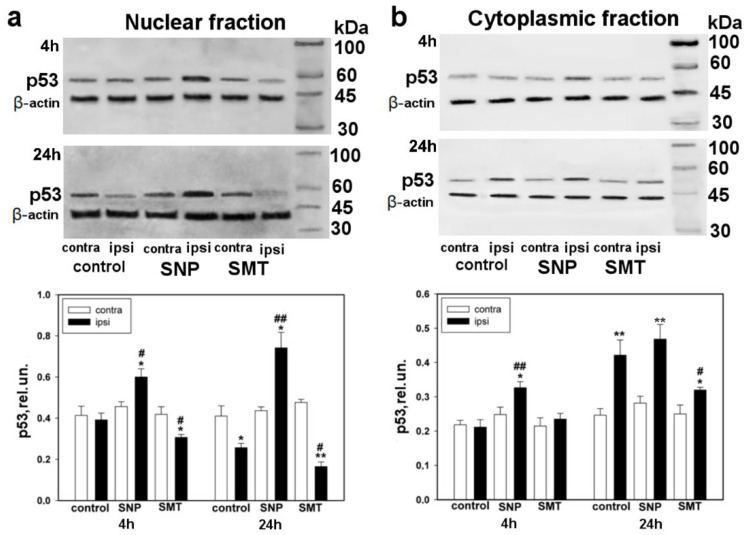
Western blot analysis. The effect of the NO donor (SNP) and the iNOS selective inhibitor (SMT) of p53 expression in the ipsilateral and contralateral DRGs, 4 h and 24 h after sciatic-nerve transection. (**a**) Nuclear fraction; (**b**) cytoplasmic fraction. One-way ANOVA. Mean ± SEM. *n* = 6. * *p* < 0.05, ** *p* < 0.01—ipsilateral DRG with respect to the contralateral DRG of the same animal; # *p* < 0.05, ## *p* < 0.01—ipsilateral DRG with respect to the ipsilateral DRG of the control group animal, within the framework of the same time interval after axotomy.

Western blot analysis confirmed the data of the immune-fluorescent microscopy. For example, it was found out that the level of p53 was increased in the nuclear fraction 4 h after axotomy and was even more increased 24 h after axotomy in the ipsilateral ganglia of rats treated with SNP in comparison with the contralateral DGRs of the experimental group (one-way ANOVA with a Dunnett’s post hoc test, *p* < 0.05, F_1,10_ = 9.701, Shapiro–Wilk test *p* = 0.128, Brown–Forsythe test *p* = 0.532 and Kruskal–Wallis H-test, *p* < 0.05, Shapiro–Wilka test *p* < 0.05, respectively) and the ipsilateral ganglia of the control group (one-way ANOVA with a Dunnett’s post hoc test, *p* < 0.05, F_1,10_ = 16.098, Shapiro–Wilk test *p* = 0.767, Brown–Forsythe test *p* = 0.819 and Kruskal–Wallis H-test, Shapiro–Wilk test *p* < 0.01, respectively) (Figure 2a). The opposite dynamics was observed in the presence of SMT, the selective inhibitor of iNOS. It was shown that in the control group the level of p53 decreased by almost two times in the nuclear fraction of axotomized DRG, 24 h after axotomy, in comparison with the opposite undamaged ganglion (one-way ANOVA with a Dunnett’s post hoc test, *p* < 0.05, F_1,10_ = 8.058, Shapiro–Wilk test *p* = 0.117, Brown–Forsythe test *p* = 0.254) (Figure 2a).

In the cytoplasmic fraction of the ipsilateral ganglia, which were obtained from animals treated with SNP, the level of p53 increased after 4 h, and by even more after 24 h, after axotomy, in comparison with the contralateral DRGs of the experimental group (one-way ANOVA with a Dunnett’s post hoc test, *p* < 0.05, F_1,10_ = 7.998, Shapiro–Wilk test *p* = 0.329, Brown–Forsythe test *p* = 0.658 and one-way ANOVA with a Dunnett’s post hoc test, *p* < 0.01, F_1,10_ = 15.610, Shapiro–Wilk test *p* = 0.571, Brown–Forsythe test *p* = 0.340, respectively) and the ipsilateral DRGs of the control group of animals (one-way ANOVA with a Dunnett’s post hoc test, *p* < 0.01, F_1,10_ = 16.943, Shapiro–Wilk test *p* = 0.968, Brown–Forsythe test *p* = 0.7) (Figure 2b). Introduction of SMT led to the decrease in the level of p53, 24 h after axotomy, in comparison with the axotomized DRGs of the control animals (one-way ANOVA with a Dunnett’s post hoc test, *p* < 0.05, F_1,10_ = 5.144, Shapiro–Wilk test *p* = 0.113, Brown–Forsythe test *p* = 0.062). In the cytoplasmic fraction of axotomized DRG, the level of p53 was increased by almost two times, as compared with the opposite undamaged DRG, 24 h after axotomy (one-way ANOVA with a Dunnett’s post hoc test, *p* < 0.01, F_1,10_ = 13.312, Shapiro–Wilk test *p* = 0.785, Brown–Forsythe test *p* = 0.236) (Figure 2b).

**Figure 3:**
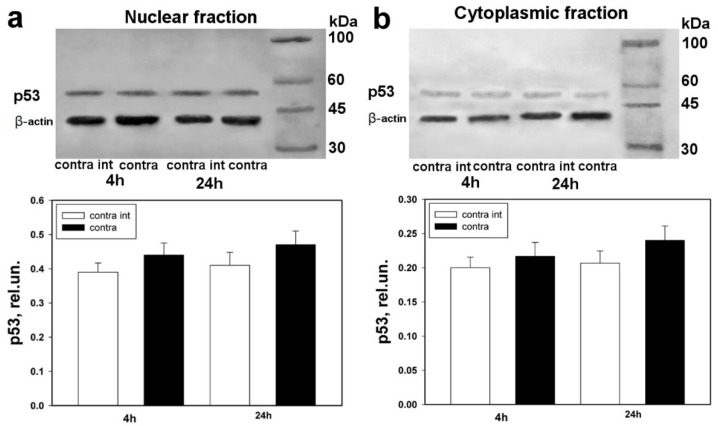
Western blot analysis. Expression of p53 in contralateral DRGs of the control group of animals, which underwent right-side sciatic-nerve transection followed by administration of physiological solution, in comparison with left-side DRGs of intact animals, to which only physiological solution was administered after 4 h and 24 h. (**a**) Nuclear fraction; (**b**) cytoplasmic fraction. One-way ANOVA. Mean ± SEM. *n* = 6.

Comparative Western blot analysis of p53 expression was carried out for contralateral DRGs of control animals, which underwent right-side sciatic-nerve transection, with respect to the left-side DRGs of intact animals, in order to exclude the influence of the stress factor (Figure 3a,b).

Western blot analysis did not reveal significant difference in the level of p53 expression in contralateral DRGs of control animals, which underwent right-side sciatic-nerve transection followed with administration of physiological solution, with respect to the level of p53 expression in left-side DRGs of intact animals, to which only physiological solution was injected. Differences in the level of p53 expression were observed neither in the nuclear nor in the cytoplasmic fraction (Figure 3a,b).

### 3.2. Upregulation of iNOS in the Nuclear and Cytoplasmic Fractions of DRGs at Different Time Intervals after Sciatic-Nerve Transection

The iNOS, which is capable of generating high level of NO, is known to play a considerable role in a variety of pathological processes [13]. It is known that trauma of peripheral nerves may induce the expression of iNOS [14]. However, neither expression nor localization of iNOS in nervous-tissue cells after axotomy are well studied. Thereby, we performed Western blot analysis of the iNOS expression in both nuclear and cytoplasmic fractions of DRGs, which were obtained from rats after sciatic-nerve transection.

**Figure 4:**
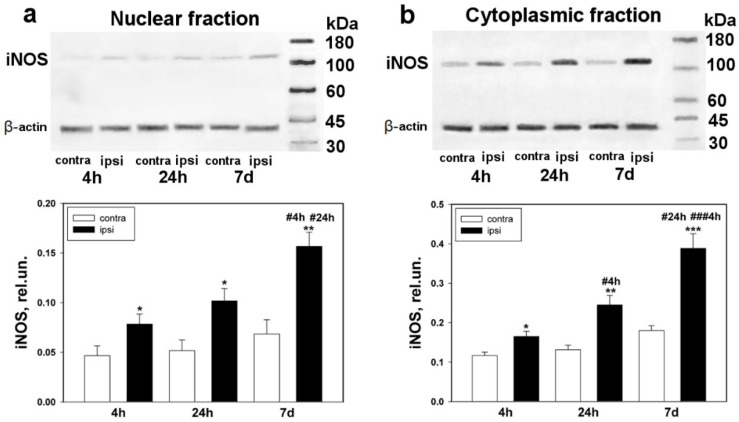
Western blot analysis. The expression of iNOS in the ipsilateral and contralateral DRGs 4 h, 24 h, and 7 days after sciatic-nerve transection. (**a**) Nuclear fraction; (**b**) cytoplasmic fraction. One-way ANOVA. Mean ± SEM. *n* = 6. * *p* < 0.05, ** *p* < 0.01, *** *p* < 0.001—ipsilateral DRG with respect to the contralateral DRG of the same animal; # *p* < 0.05, ### *p* < 0.001—ipsilateral DRG with respect to the ipsilateral DRG, at different time intervals (with respect to 4 h and 24 h).

The Western blot analysis showed that iNOS is located mostly in the cytoplasmic rather than nuclear fraction of DRG (Figure 4a,b). In undamaged DRGs, the iNOS expression was observed neither in the nuclear nor in the cytoplasmic fraction. However, after sciatic-nerve transection, upregulation was observed after 4 and 24 h and reached its maximal level seven days after axotomy (Figure 4a,b). For example, it was shown that the level of iNOS was increased in both the nuclear and cytoplasmic fractions of the ipsilateral ganglion by 70% (one-way ANOVA with a Dunnett’s post hoc test, *p* < 0.05, F_1,10_ = 5.0, Shapiro–Wilk test *p* = 0.346, Brown–Forsythe test *p* = 0.535) (Figure 4a) and 40% (One-Way ANOVA with a Dunnett’s post hoc test, *p* < 0.05, F_1,10_ = 9.622, Shapiro–Wilk test *p* = 0.861, Brown–Forsythe test *p* = 0.246) (Figure 4b), respectively, with respect to the control DRG, 4 h after axotomy, whereas after 24 h the level of iNOS increased by two times (Kruskal–Wallis H-test, *p* < 0.05, Shapiro–Wilk test *p* = 0.531, Brown–Forsythe test *p* < 0.050) (Figure 4a) and by 86% (one-way ANOVA with a Dunnett’s post hoc test, *p* < 0.01, F_1,10_ = 17.649, Shapiro–Wilk test *p* = 0.794, Brown–Forsythe test *p* = 0.080) (Figure 4b), respectively.

In the nuclear and cytoplasmic fractions of the seven-day-old axotomized DRG, the expression of iNOS was increased by 2.1 times (one-way ANOVA with a Dunnett’s post hoc test, *p* < 0.01, F_1,10_ = 18.852, Shapiro–Wilk test *p* = 0.537, Brown–Forsythe test *p* = 0.890) (Figure 4a) and by 2.2 times (one-way ANOVA with a Dunnett’s post hoc test, *p* < 0.0, F_1,10_ = 28.012, Shapiro–Wilk test *p* = 0.051, Brown–Forsythe test *p* = 0.111) (Figure 4b), respectively, as compared with the undamaged ganglion.

### 3.3. The NO-Dependent Activation of Neuron and Glial Cell Death by NO Donors and Cytoprotector Effect of the Selective Inhibitor of iNOS after Axotomy

Presently, there is no common opinion on the role of NO in death and survival of neurons and glial cells after axotomy [5,6,7,8,9,10,11]. Therefore, we decided to study the effect of the NO donor, sodium nitroprusside, and the selective inhibitor of iNOS, S-methylthiourea hemisulfate, on the death of neurons and glial cells of DRGs after sciatic-nerve transection.

**Figure 5:**
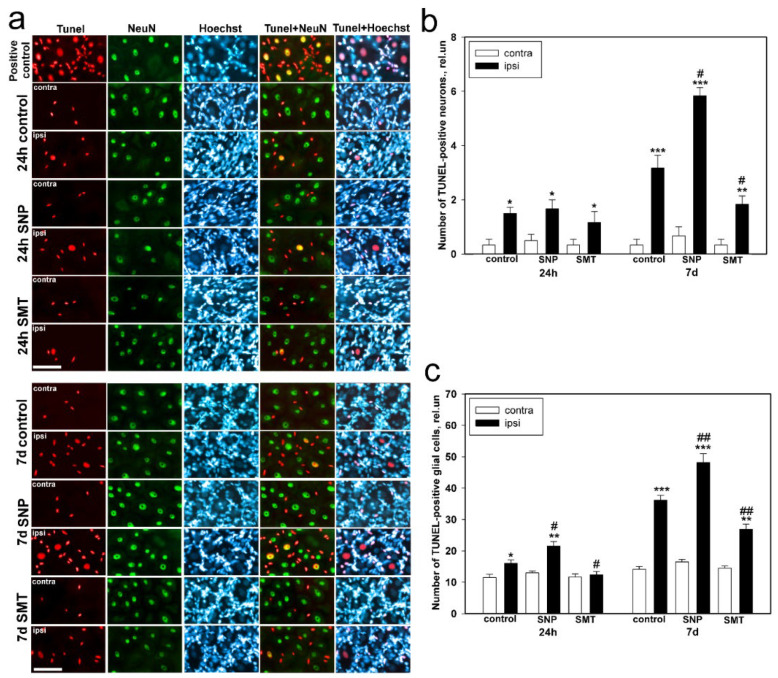
Immune fluorescent microscopy. (**a**) The DRG sections obtained from the control group of animals, which were treated with physiological solution, and the experimental groups of animals, which were treated with the NO donor (SNP) or the iNOS selective inhibitor (SMT), 24 h and 7 days after sciatic-nerve transection were stained with TUNEL, and the DRG sections were stained with TUNEL in the presence of benzonase nuclease (positive control). The scale section is 50 μm. (**b**) The number of TUNEL-positive DRG neurons in the control and experimental groups of animals, 24 h and 7 days after sciatic-nerve transection. (**c**) The number of TUNEL-positive glial cells in the control and experimental groups of animals, 24 h and 7 days after sciatic-nerve transection. Ipsi—the axotomized ipsilateral ganglion; contra—the contralateral ganglion; TUNEL—nuclear marker of cell apoptosis (red fluorescence); NeuN—the neuronal nuclei marker (green fluorescence); TUNEL + NeuN and TUNEL + Hoechst—overlap; Hoechst—fluorescence of the Hoechst 33342, which allows visualization of both neural and glial cell nuclei. One-way ANOVA. Mean ± SEM. *n* = 6. * *p* < 0.05, ** *p* < 0.01, *** *p* < 0.001—ipsilateral DRG with respect to the contralateral DRG of the same animal; # *p* < 0.05, ## *p* < 0.01—ipsilateral DRG with respect to the ipsilateral DRG of the control group animal, within the framework of the same time interval after axotomy.

The TUNEL analysis of the contralateral DRGs, which did not undergo axotomy, revealed few TUNEL-positive neurons in 1 day and 7 days after sciatic-nerve transection (Figure 5a). After 24 h after axotomy, the ipsilateral ganglia of both the control and experimental groups demonstrated equal confident tendency to the increase in apoptosis of neurons, which increased significantly 7 days after trauma (Figure 5b).

In 7 days after sciatic-nerve transection, the number of TUNEL-positive neurons in the ipsilateral ganglia of the control groups and the experimental group, which underwent treatment with SNP (NO donor) and SMT (iNOS inhibitor), increased by 7.5 times (from 0.4 ± 0.2 to 3 ± 0.5 relative units) (one-way ANOVA with a Dunnett’s post hoc test, *p* < 0.001, F_1,10_ = 29.49, Shapiro–Wilk test *p* = 0.194, Brown–Forsythe test *p* = 0.209), by 10 times (from 0.6 ± 0.3 to 5.8 ± 0.7 relative units) (one-way ANOVA with a Dunnett’s post hoc test, *p* < 0.001, F_1,10_ = 129.865, Shapiro–Wilk test *p* = 0.074, Brown–Forsythe test *p* = 0.563), and by 6 times (from 0.3 ± 0.1 to 1.8 ± 0.3 relative units) (one-way ANOVA with a Dunnett’s post hoc test, *p* < 0.01, F_1,10_ = 16.200, Shapiro–Wilk test *p* = 0.386, Brown–Forsythe test *p* = 0.599), respectively, in comparison with the contralateral DRGs (Figure 5b). The number of the TUNEL-positive neurons in the ipsilateral DRGs, which were treated with SNP and the iNOS inhibitor, was increased in the first case by 1.8 times (one-way ANOVA with a Dunnett’s post hoc test, *p* < 0.001, F_1,10_ = 22.069, Shapiro–Wilk test *p* = 0.456, Brown–Forsythe test *p* = 0.401) and decreased in the second case by 1.7 times (one-way ANOVA with a Dunnett’s post hoc test, *p* < 0.05, F_1,10_ = 5.517, Shapiro–Wilk test *p* = 0.456, Brown–Forsythe test *p* = 0.401) in comparison with the ipsilateral DRG of the control group, respectively (Figure 5b).

The glial cells underwent stronger cell death than neurons (Figure 5a,b). The TUNEL-positive glial cells were found in both control contralateral and axotomized ganglion in higher number than neurons (Figure 5a,c). In 24 h after axotomy, the death of glial cells in the ipsilateral DRG of the control group and the experimental group, which was treated with SNP, increased by 33% (from 12 ± 1 to 16 ± 1 relative units) (one-way ANOVA with a Dunnett’s post hoc test, *p* < 0.05, F_1,10_ = 8.265, Shapiro–Wilk test *p* = 0.819, Brown–Forsythe test *p* = 0.857) and by 62% (from 13 ± 1.4 to 21 ± 3 relative units) (Kruskal–Wallis H-test, *p* < 0.01, Shapiro–Wilk test *p* = 0.998, Brown–Forsythe test *p* < 0.05), respectively, as compared with the contralateral DRGs (Figure 5c). Administration of the iNOS inhibitor led to the decrease in cell death of glial cells of the axotomized DRGs in comparison with the contralateral ganglia, and the number of apoptotic cells was 25% lower (one-way ANOVA with a Dunnett’s post hoc test, *p* < 0.05, F_1,10_ = 5.354, Shapiro–Wilk test *p* = 0.195, Brown–Forsythe test *p* = 1.0), in comparison with the ipsilateral DRGs of the control group (Figure 5c). The comparative analysis of glial-cell death in the ipsilateral DRGs of animals treated with SNP and SMT, which was carried out between the groups with respect to the ipsilateral DRGs of the control animals, showed the increase in the number of death cells as 30% (one-way ANOVA with a Dunnett’s post hoc test, *p* < 0.05, F_1,10_ = 8.941, Shapiro–Wilk test *p* = 0.877, Brown–Forsythe test *p* = 0.441) and the decrease as 25% (one-way ANOVA with a Dunnett’s post hoc test, *p* < 0.05, F_1,10_ = 5.354, Shapiro–Wilk test *p* = 0.195, Brown–Forsythe test *p* = 1.0), respectively (Figure 5c).

A considerable increase in the number of TUNEL-positive glial cells in the ipsilateral DRGs of all groups of animals was observed 7 days after sciatic-nerve transection (Figure 5a,c). For example, the level of apoptosis of glial cells in the ipsilateral DRGs of the control group and the experimental group, which underwent treatment with SNP and SMT, increased by 2.6 times (from 14 ± 1 to 36 ± 2 relative units) (one-way ANOVA with a Dunnett’s post hoc test, *p* < 0.001, F_1,10_ = 161.933, Shapiro–Wilk test *p* = 0.818, Brown–Forsythe test *p* = 0.404), by three times (from 16 ± 2 to 48 ± 3 relative units) (one-way ANOVA with a Dunnett’s post hoc test, *p* < 0.001, F_1,10_=121.141, Shapiro–Wilk test *p* = 0.519, Brown–Forsythe test *p* = 0.106) and by 1.9 times (from 14 ± 2 to 27 ± 3 relative units) (Kruskal–Wallis H-test, *p* < 0.01 Shapiro–Wilk test *p* = 0.995, Brown–Forsythe test *p* < 0.050), respectively, in comparison with the contralateral DRGs (Figure 5c). At the same time, the level of cell death in the ipsilateral DRGs of the groups treated with SNP and SMT increased by 33% (one-way ANOVA with a Dunnett’s post hoc test, *p* < 0.01, F_1,10_ = 14.513, Shapiro–Wilk test *p* = 0.663, Brown–Forsythe test *p* = 0.291) and decreased by 25% (one-way ANOVA with a Dunnett’s post hoc test, *p* < 0.01, F_1,10_ = 17.007, Shapiro–Wilk test *p* = 0.683, Brown–Forsythe test *p* = 0.426), respectively, in comparison with the ipsilateral DRGs of the control animals (Figure 5c).

**Figure 6:**
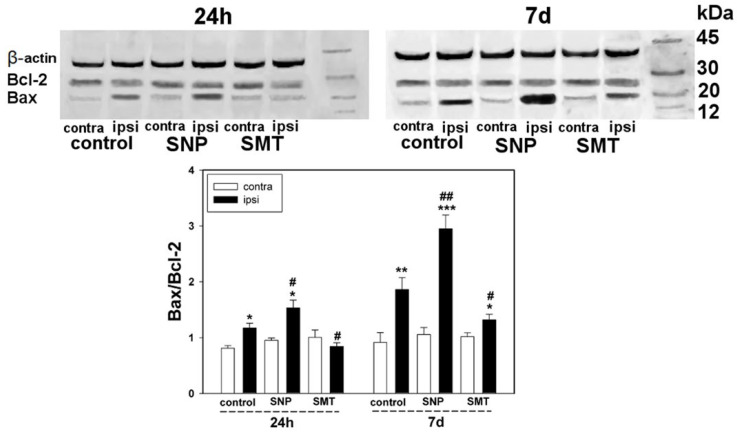
Western blot analysis. The effect of NO donor (SNT) and selective inhibitor (SMT) of iNOS on the Bax–Bcl-2 ratio in the ipsilateral DRG and contralateral DRG, 24 h and 7 days after sciatic-nerve transection. One-way ANOVA. M ± SEM. *n* = 6. * *p* < 0.05, ** *p* < 0.01, *** *p* < 0.001—ipsilateral DRG with respect to contralateral DRG of the same animal # *p* < 0.05, ## *p* < 0.01—ipsilateral DRG with respect to ipsilateral DRG of the control group animal, within the same temporal interval after axotomy.

Data on the NO-dependent cell death in DRGs after axotomy, obtained by the method of TUNEL, were also confirmed by Western blot analysis of the Bax–Bcl-2 protein ratio. It is known that Bax activates cell death, inducing permeabilization of the mitochondrial membranes [40]. The Bcl-2 protein is the molecular antagonist of the proapoptotic effects of Bax [41]. Balance between the levels of these proteins significantly affects cell fate, leading to either survival or death [40]. It was shown that sciatic-nerve transection resulted in a 37% and two times increase in the Bax–Bcl-2 index in the cells of injured ganglia, in comparison with the contralateral ganglia, 24 h and 7 days after injury, respectively (one-way ANOVA with a Dunnett’s post hoc test, *p* < 0.05, F_1,10_ = 13.512, Shapiro–Wilk test *p* = 0.702, Brown–Forsythe test *p* = 0.422) (one-way ANOVA with a Dunnett’s post hoc test, *p* < 0.01, F_1,10_ = 11.685, Shapiro–Wilk test *p* = 0.071, Brown–Forsythe test *p* = 0.690) (Figure 6).

This negative effect was increased in axotomized DRGs after introduction of the SNP NO donor. Indeed, the Bax–Bcl-2 index was increased by 60% in ipsilateral DRGs with respect to the contralateral DRGs of the animals, which received SNP 24 h (Kruskal–Wallis H-test, *p* < 0.05, Shapiro–Wilk test *p* < 0.050, Brown–Forsythe test *p* = 0.199) that was increased three times in 7 days (one-way ANOVA with a Dunnett’s post hoc test, *p* < 0.001, F_1,10_ = 46.975, Shapiro–Wilk test *p* = 0.171, Brown–Forsythe test *p* = 0.170), respectively (Figure 6). Moreover, confident differences were observed with respect to ipsilateral ganglia of the control group as well. In the ipsilateral DRGs of the animals that received SNP, the Bax–Bcl-2 index was increased with respect to the ipsilateral DRGs of the control animals by 40% after 24 h (one-way ANOVA with a Dunnett’s post hoc test, *p* < 0.05, F_1,10_ = 5.044, Shapiro–Wilk test *p* = 0.186, Brown–Forsythe test *p* = 0.559) and by 62% after 7 days (one-way ANOVA with a Dunnett’s post hoc test, *p* < 0.01, F_1,10_ = 11.055, Shapiro–Wilk test *p* = 0.104, Brown–Forsythe test *p* = 0.714), respectively (Figure 6).

Inhibition of iNOS with SMT revealed a cytoprotector effect, which manifested as a decrease in proapoptotic activity after the trauma. It was shown that the Bax–Bcl-2 index decreased in ipsilateral DRGs, with respect to the ipsilateral DRGs of the control animals, by 33% (one-way ANOVA with a Dunnett’s post hoc test, *p* < 0.05, F_1,10_ = 9.372, Shapiro–Wilk test *p* = 0.309, Brown–Forsythe test *p* = 0.754) and 27% (one-way ANOVA with a Dunnett’s post hoc test, *p* < 0.05, F_1,10_ = 5.236, Shapiro–Wilk test *p* = 0.411, Brown–Forsythe test *p* = 0.287), after 24 h and 7 days, respectively (Figure 6). It is noteworthy that changes in the Bax–Bcl-2 ratio in injured ganglia was mostly due to the increase in the level of Bax rather than decrease in the level of Bcl-2.

## 4. Discussion

It is known that NO may regulate the expression of a number of proteins, including p53. Regulation of p53 expression through the NO-dependent signaling cascades has been studied in a variety of experimental models, using the NO donors and inhibitors of NOS [17,18,36,37,38,39]. However, mechanisms of NO-dependent regulation of p53 in the peripheral nervous system following axotomy has been studied to a very limited extent.

**Figure 7:**
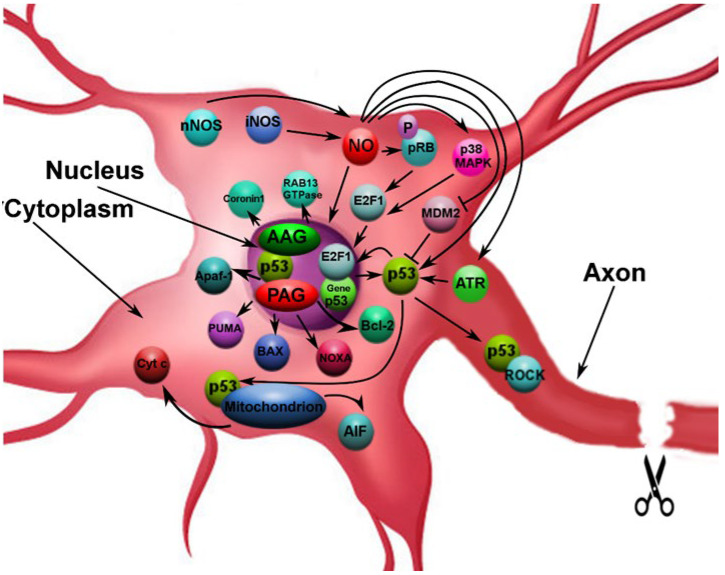
Scheme of NO-dependent realization of pro- and anti-apoptotic p53-signaling pathways in axotomized neurons. The NO, which is generated by iNOS and nNOS in result of axonal stress, may regulate p53 expression and localization by a variety of direct and indirect ways. Note: PAG—pro-apoptotic genes; AAG—anti-apoptotic genes.

In the present study, we have shown that NO plays an important role in the regulation of the expression and localization of p53 under the conditions of axotomy. The NO-dependent upregulation of p53, which we observed in neurons and glial cells of DRGs, 4 h and 24 h after sciatic-nerve transection, in the presence of NO donors, is in agreement with previous studies, which showed NO to be a powerful inductor of p53 expression [16,17,18,36,37,39]. The increase in the level of p53 in the nuclei and cytoplasm of neurons, 4 h after axotomy, may be considered as the evidence of either activation of the p53 gene transcription or the NO-dependent mechanism of the decrease in the p53 degradation rate. For example, it is known that NO, at early stages, induces the decrease in the level of Mdm2 via the decrease in its ubiquitination and the following degradation of p53 in proteasomes [18]. The effect of NO-dependent upregulation of the p53 gene may be achieved via activation of E2F1, the upregulation of which was shown in our previous studies. It is known that NO induces the increase in the level of E2F1 via the hyperphosphorylation and inactivation of pRb. It also increases the DNA-binding capacity of the E2F1 via activation of the p38 MAPK [42]. The E2F1, in turn, works as the transcription factor for a number of genes including p53 (Figure 7) [43,44].

The NO-induced nuclear deposition of p53 in neurons and glial cells was expressed most strongly 24 h after sciatic-nerve transection. No nuclear–cytoplasmic translocation of p53, which was observed in the axotomized neurons of the control group, was found in the neurons of the animals treated with the NO donors. The level of p53 in the cytoplasm of ipsilateral DRG neurons of animals treated with the NO donor was similar to that in the ipsilateral DRG of the control group, 24 h after axotomy. At the same time, the level of p53 in the nuclei was almost three times higher than that in the control group, pointing at both an increased synthesis of p53 and its depositing in the neuronal nucleoplasm that was induced by the excess of NO. The glial cells of axotomized DRGs of animals treated with NO donor were also characterized by an increased level of p53, though it was lower than in the damaged neurons. These data are consistent with the data of previous studies, which demonstrated that, at early stages after stress, the NO donors induce the decrease in the level of Mdm2, the key enzyme, which is responsible for ubiquitination and the following degradation of p53. The NO was also shown to inhibit nuclear export of p53 via the serine/threonine protein kinase (ATP), which phosphorylates the Ser-15 residues and inactivates signals of nuclear export of p53, resulting in the depositing of p53 in the nucleoplasm. It is known that the NO-induced nuclear accumulation of p53 may lead to apoptosis [17,18,37].

The NO-induced nuclear deposition of p53 in neurons and glial cells was expressed most strongly at the late stages after trauma, to be more exact, 24 h after sciatic-nerve transection. No nuclear–cytoplasmic translocation of p53, which was observed in the axotomized neurons of the control group, was found in the neurons of the animals treated with the NO donors. The level of p53 in the cytoplasm of ipsilateral DRG neurons of animals treated with the NO donor was similar to that of the ipsilateral DRG in the control group, 24 h after axotomy. At the same time, the level of p53 in the nuclei was almost three times higher than that in the control group, pointing at both increased synthesis of p53 and its depositing in the neuronal nucleoplasm, which was induced by excess amounts of NO. The glial cells of axotomized DRGs of animals treated with NO donor were also characterized by increased level of p53, though it was lower than in the damaged neurons. These data are consistent with the data of previous studies, which demonstrated that at early stages after stress the NO donors induce the decrease in the level of Mdm2, the key enzyme, which is responsible for ubiquitination and the following degradation of p53. The NO was also shown to inhibit nuclear export of p53 via the serine/threonine protein kinase (ATP), which phosphorylates the Ser-15 residues and inactivates signals of nuclear export of p53, resulting in depositing of p53 in nucleoplasm. It is known that the NO-induced nuclear accumulation of p53 may lead to apoptosis [17,18,37].

The p53-modulating effects of NO may be achieved through the products of its active metabolism during the development oxidative stress in axotomized neurons. It is known that sodium nitroprusside is a powerful donor of NO, which induces nitrosative stress via the release of NO. The latter may interact with oxygen, as well as superoxide anion-radical and transition metals, to form the reactive nitrogen species, the most powerful of which is peroxynitrite (ONOO^−^). This radical provides nitration of amino acids, such as, for example, tyrosine, and oxidizes a variety of biomolecules, including proteins, lipids, amino acids, and nucleotides [45]. It was shown that the nitration of Tyr-327 in the tetramerization domain of p53 induces its nuclear depositing [46]. Moreover, the nitration of Tyr-181 residue in the IκBα induces activation of the NF-κB transcription factor [46], which is particularly responsible for the expression of iNOS [47]. The resulting hyperproduction of NO, which is generated by the iNOS at the background of endogenous NO, leads to the increase in the nitrosative stress, closing the feedback circuit of the NO-dependent signaling pathway in regulation of p53 expression (Figure 7).

It is noteworthy that NO may provide its effects on the p53 level both in the cytoplasm and directly in neuronal nuclei. The NO being a lypophilic molecule, it may pass easily through biological membranes. However, the diffuse distribution of NO may be limited by its high reactivity. In our studies, we observed both the directly nuclear and cytoplasmic localization of iNOS under the conditions of axotomy. It is known that all isoforms of iNOS may penetrate the nucleus from cytoplasm under stress conditions [48]. Several studies revealed nuclear localization of iNOS [49,50], as well as a partially constitutive type of its expression [51]. The penetration of iNOS into the karyoplasm may represent one more mechanism of regulation of nuclear export of p53 by inducible NO. Upregulation of iNOS, which was revealed by Western blot, was observed in the early stages after axotomy and reached its maximal values 7 days after trauma. These data are consistent with previous studies, which showed rapid expression of iNOS that reached its maximum 7 days after axotomy [52].

In contrast to nNOS and eNOS, the inhibition of iNOS, which is one of the key enzymes of the inflammation system, generating a high level of NO, led to the downregulation of p53 in the neuronal, but not in the glial cell nuclei, 4 h and 24 h after axotomy. This may be due to the delay in the expression of iNOS in glial cells, whereas in neurons it is expressed constitutively.

Nuclear–cytoplasmic translocation of p53 in the axotomized neurons in the absence of the NO donor points at the realization of p53 transcriptionally independent signaling pathways under the conditions of axonal stress. It is known that p53 may induce apoptosis through binding to mitochondria, distortion of bioenergetics processes and release of cytochrome *c* and AIF, which induce activation of caspase 3, the key pro-apoptotic protease (Figure 7) [53]. This, finally, leads to apoptosis [19,54]. The p53 may however participate in the processes of neuron survival. It may direct growth of axons via regulation of expression of the actin-binding protein Coronin 1b and small GTPase Rab13, which play the key role in the cytoskeleton remodeling (Figure 7) [27]. These functions are mediated by acetylation of p53 that has been recently shown to induce a new transcription module, which includes p53, histon acetyltransferases (HAT), CREB-binding protein/p300 (CBP/p300) and p300-CBP binding factor (P/CAF), which upregulate the GAP-43 protein that is involved into the axon growth [55,56]. Moreover, the phosphorylated p53 may directly interact with the Rho-kinase (ROCK) in the axon, which sustains axon growth via regulation of the actin system and microtubules (Figure 7) [57]. Apparently, the observed after axotomy accumulation of p53 the cytoplasm of neurons may be considered to be a molecular-cellular event, which precedes the p53 transportation into the nucleus, besides the pro-apoptotic mechanism. However, the DRG neurons are unipolar. In the sciatic nerve their processes divide into the afferent and efferent branches. Inside the ganglion the processes of these neurons are not found, providing few reasons to believe that the transport of p53 takes place along the axons.

The observed nuclear expression of p53 in glial cells, which remained after axotomy, points at the significant difference in realization of the p53 signaling cascade in glial cells from that in the neurons, in which nuclear–cytoplasmic translocation of p53 was observed. It is known that p53 of astrocytes plays a fundamental role in pathogenesis of a variety of neurodegenerative diseases [23]. We have also studied the role of NO in the survival and death of neurons and glial cells in DRGs after sciatic-nerve transection. Axotomy is a powerful type of stress for both neurons and surrounding glia, which initiates the cascade of molecular and cellular processes, resulting in either death or survival of these cells. It is known that NO may provide both neuroprotector and neurotoxic effects. For example, it was shown that deficiency of nNOS in mice correlates with increased cell death in DRGs in the spinal cord after transection of peripheral nerves [58,59]. However, several studies point to the role of NO in negative regulation of survival of neurons and glial cells after neurotrauma [60,61,62]. It may induce necrosis via exhaustion of energy because of the inhibition of oxidative phosphorylation, glycolysis, Ca^2+^ excitotoxicity, increase in mitochondrial membrane permeability, etc. The NO may induce mechanisms of apoptotic cell death via activation of p53, p38 MAPK, and stress of endoplasmic reticulum. However, NO may also provide cytoprotective effects via the decrease in membrane permeability and activation of Akt. It also provides vasodilation via the cGMP-dependent signaling pathway [63]. Nevertheless, the role of NO in neural and glial-cell death and survival after axotomy is contradictory.

In the present study, we showed that NO is responsible for neural and glial-cell death in DRGs of rats. The level of cell death of glial cells was considerably higher in comparison with neurons of both axotomized and control ganglia that is consistent with the data of previous studies [64,65]. The level of death of neurons and glial cells increased in axotomized DRGs of animals, which underwent treatment with the NO donor after axotomy. Administration of the selective inhibitor of iNOS led to the cytoprotective effect. It is known that the iNOS is expressed in both glial cells [66] and neurons [67]. Upregulation of iNOS in glial cells was observed after the influence of different stimuli, such as ischemia, treatment with lipopolysaccharides and cytokines, as well as after the peripheral nerve transection [68]. In our study, the expressed neuronal and glial apoptosis, which was observed on the 7th day after axotomy, coincided with the peak of the iNOS expression. The data obtained show that NO, and particularly its inducible hyperproduction, plays an important role in neural and glial-cell death after axotomy. This is consistent with the data of previous studies, which showed the iNOS expression to be one of the key mechanisms of cell death under the stress conditions [12,69,70], including axotomy [39,68].

Mechanisms of apoptotic death of neurons may be achieved through the satellite glia, which is activated by inducible hyperproduction of NO. Activation of phagocytic NADPH-oxidase and expression of iNOS in glial cells lead to apoptosis via the production of ONOO^−^. On the other hand, the NO, which is produced by iNOS, induces neural-cell death via the inhibition of cytochrome oxidase [12]. Moreover, neurons and glial cells may undergo apoptosis due to the activation of p53-mitochondrial mechanism. Our studies showed activation of the p53 signaling pathway in neurons and glial cells of both vertebrate and invertebrate animals in response to the damage of axons [28,29]. In turn, p53 and NF-κB regulate each other either positively or negatively, depending on the intracellular conditions [71]. The NF-κB may, in turn, upregulate the iNOS (Figure 7) [47,72].

Therefore, we have shown that NO, which is particularly generated by the iNOS, may play a role in regulation of p53 in neurons and glial cells under the conditions of axonal stress. The NO is responsible for accumulation of p53 in the nuclei of these calls after trauma of the nerve. Sciatic-nerve transection induced cell death of neurons and glial cells, and treatment with the NO donor enhanced this process. On the other hand, treatment with the iNOS inhibitor provided opposite effect. Death of neural and glial cells after axotomy may be achieved via the NO-dependent mechanisms of regulation of p53 expression.

## 5. Conclusions and Future Decisions

The results of our study showed that:

1. Axotomy induces nuclear–cytoplasmic redistribution of p53 in DRG neurons, 24 h after trauma.
2. The NO donor (SNP) induces nuclear deposition of p53 in neurons and glial cells of DRGs, 4 h and 24 h after axotomy.
3. The selective inhibitor of inducible NO-synthase (SMT) induces decrease in the level of p53 in DRG neurons, 4 h and 24 h after axotomy.
4. Axotomy induces increase in the level of iNOS in both nucleus and cytoplasm of DRG cells, 4 h, 24 h, and 7 days after trauma.
5. Axotomy increases the level of neural and glial-cell death, 24 h and 7 days after trauma.
6. The NO donor SNP increases neural and glial-cell death, 24 h and 7 days after axotomy.
7. The selective inhibitor of inducible NO-synthase SMT decreases neural and glial-cell death, 24 h and 7 days after trauma.
8. Axotomy increases the Bax–Bcl-2 index in DRG cells, 24 h and 7 days after trauma.
9. The NO donor (SNP) significantly increases the Bax–Bcl-2 index in DRG cells, 24 h and 7 days after axotomy.
10. The selective inhibitor of inducible NO-synthase (SMT) decreases the Bax–Bcl-2 index in DRG cells, 24 h and 7 days after axotomy.

The perspective goal of this research is the detailed study of NO-dependent effects on different proteins, which possess pro- and antiapototic activity, in neurons and glial cells under the axonal-stress conditions. The data obtained provide the impact into the fundamental understanding of signaling mechanisms involved into the survival and death of neurons and glial cells after neurotrauma, which may help develop effective neuroprotector medications.

## Data Availability

Not applicable.
